# Comparison of Microalbuminuria Status in Type 2 Diabetes Mellitus Patients With Chronic Periodontitis: A Cross-Sectional Study

**DOI:** 10.7759/cureus.27383

**Published:** 2022-07-28

**Authors:** Deepa K Nair, B. Shanthi, P. Nandu Baby

**Affiliations:** 1 Biochemistry, Bharath Institute of Higher Education and Research, Tambaram, IND; 2 Biochemistry, Sree Balaji Medical College and Hospital, Chennai, IND; 3 Biochemistry, Premier Healths, Edappal, IND

**Keywords:** periodontitis, nephelometry, microalbuminuria, hba1c, diabetes, cpi

## Abstract

Background

Diabetes has increased the risk for various other ailments in various organs of the body. This can be contributing to periodontitis also as it is the sixth complication related to diabetes mellitus. There is a bidirectional relationship between both. Given the high global prevalence of type-2 diabetes mellitus (T2DM) with periodontitis, it is of great importance to determine the link between periodontitis and microalbuminuria in T2DM patients, which shows early renal disease.

Methodology

In the present study, a total of 500 patients having T2DM were assessed for periodontitis using Community Periodontal Index (CPI). Anthropometric and biochemical measurements were obtained. Blood samples were estimated for glycemic control tests such as fasting plasma glucose (FPG), glycated hemoglobin (HbA1c), and lipid profile. The subjects who participated in the study were categorized into three groups depending on the albuminuria level. The data were tabulated and analyzed using SPSS Statistics software (IBM Corp., Armonk, USA).

Results

Out of 500 T2DM subjects, 342 subjects had periodontitis. A statistically significant difference was found in FPG, HbA1c, total cholesterol (TC), triglycerides, and low-density lipoprotein (LDL) between subjects with periodontitis and without periodontitis using a t-test (p = <0.001). The prevalence of normoalbuminuria, micro-, and macroalbuminuria among periodontitis patients was 24.6%, 72.8%, and 2.6% respectively, and the Chi-square analysis revealed that was highly significant. In terms of albuminuria, one-way analysis of variance (ANOVA) revealed statistically significant differences among the periodontitis subjects for the following variables: inputs such as the number of teeth, diabetes mellitus (DM) duration, the level of LDL, and also the depth of the pocket. Intergroup comparison of variables among subjects with albuminuria using the statistical test of Tukey Post Hoc found that there is a significant difference between normoalbuminuria and microalbuminuria. CPI score, tooth mobility, smoking, education level, family income, tooth brushing duration, along with the use of other dental hygiene aids was also found to be statistically significant among subjects with periodontitis.

Conclusion

The study concluded that T2DM patients had a higher incidence of microalbuminuria among individuals with periodontitis. These subjects also had significantly higher HbA1c and FPG levels than subjects with normoalbuminuria. In addition, subjects with periodontitis exhibited a significant reduction in the total teeth numbers present in the case of albuminuria. The longitudinal correlation between DM, microalbuminuria, and periodontitis could be further investigated in detail to explore possible pathways.

## Introduction

According to current estimates, 463 million individuals in 2019 to 537 million in 2022 were estimated to have the world’s most common chronic disease - diabetes mellitus (DM). By 2045, there will likely be 700 million individuals worldwide residing with diabetes, with the prevalence expected to rise by approximately 50% during the following years [1]. Resisting insulin production affects the naturally occurring insulin availability within the codocytes, which is the cause of non-insulin-dependent type 2 DM (T2DM). Due to the delayed onset and frequent lack of symptoms, type 2 diabetic individuals may go years without diagnosis and treatment [2].

Patients with diabetes are much more likely to experience other health problems, like diabetic retinopathy, diabetic nephropathy, cardiovascular disease, and diabetic neuropathy. In light of the fact that less than 25% of patients with diabetes would ultimately experience a revolutionary decline in kidney function, prevention of diabetes-related severe issues, particularly chronic kidney disease (CKD) and cardiovascular dysfunction, is crucial for the management of T2DM [3]. Additionally, there is evidence to suggest that diabetics have periodontitis more frequently than those without the disease [1].

The dental-related disease periodontitis and diabetes are both complex and severe diseases that have a known two-way relationship. The sixth most frequent consequence of diabetes mellitus is periodontal disease [1,2,4-6]. Poor glycemic management with glycated haemoglobin (HBA1c) levels above 7 can result in poor oral health, which increases the risk of periodontal disease and bone loss by 2.8 and 4.2-times, respectively [4]. Chronic periodontitis leads to an increase in the number of proinflammatory cytokines, which in turn causes the structural breakdown of periodontal tissues and results in tooth loss. Due to periodontitis, there is a breakdown of the periodontal ligament's collagen fibres, and a periodontal pocket forms between the gingiva and the teeth. Vascular aberrations, neutrophil dysfunction, anomalies in collagen production, and genetic predilection are some of the processes by which diabetes affects periodontal disease [7].

Albuminuria is a significant indicator of subclinical atherosclerosis, widespread microvascular dysfunction, and kidney damage, and it is linked to a higher chance of later cardiovascular morbidity in people with diabetes or hypertension, including in the common populace. It is widely known that patients with micro- or macroalbuminuria have more advanced pathology than those with a normal range of albuminuria. Microalbuminuria and periodontitis seem to be associated with systemic inflammation [8,9]. The mechanism of action of the bidirectional relationship exists due to the hyperglycemic state which leads to the accumulation of monocytes and lipids in the epithelium and connective tissue leading to more damage and collagen breakdown. In addition, microalbuminuria is implicated in the development of hypertension, atherogenic nephropathy, overt nephropathy, and eventually renal failure [9-11].

Studies show that microalbuminuria is widespread in the general populace, with a generality of 6.6% even in hypertensive or non-diabetic patients and that it also serves as a standalone dangerous factor for other types of metabolic dysfunction [11,12]. Given the high global incidence of T2DM and periodontitis, it is of great importance to determine the association between periodontitis and microalbuminuria indicating early renal pathology in patients with T2DM.

## Materials and methods

Study sample

The study comprised 500 T2DM patients who were treated in a south Kerala outpatient clinic from January 2021 to April 2022. There were 260 males and 240 females, all with similar age distribution. The samples were collected from PMS Dental College, Kerala, and permitted by the Institutional Human Ethical Committee with the reference number, Ref. No. PMS/IEC/2019-20/38. Type-2 DM diagnosed by a diabetologist within a month, having 12 or more teeth between the ages of 31 and 65 years, and being free of other clinical conditions involving proteins in urine were the inclusion criteria.Type-1 diabetic patients and individuals with a history of any other routine health issues like atherosclerosis, retinopathy, liver cirrhosis, obstructive sleep apnea, and immunologic or mental disorders were not included. Before being enrolled in the current study, informed consent was signed by all participants. The procedures and study design were evaluated and approved by a member of the Institutional Ethical Committee, who was also responsible for ensuring that the research was carried out in accordance with the Declaration of Helsinki.

Socioeconomic factors

The study participants were interviewed regarding their cigarette smoking habits, daily alcohol intake, routine exercise, education status, and monthly family income. Patients were categorized as ever smokers or non-smokers on the basis of their responses to the individual-reported valid questions. Participants who had smoked a minimum of five packets of cigarettes during their entire life were considered to be "ever-smokers". Based on the daily alcohol consumption during the last four weeks prior to the self-interview, patients were divided into non-drinkers, moderate drinkers (4 to 14 drinks per week), or strong drinkers (more than two drinks per day).

According to the IPAQ (International Physical Activity Questionnaire) short form [13], patients were deemed as frequently exercising individuals if they were engaged in moderate physical exercise five times or more in a week for more than 30 minutes per day or intense physical activity more than three times per week for more than 20 minutes per day. The number of years of schooling was used to divide the educational level into >12 years (high school/graduate) or fewer. The lower 25 percentile of the average monthly family income or greater of the study sample was considered the reference group using the Likert scale.

Anthropometric, biochemical measurements and definitions

Skilled staff members obtained anthropometric and biochemical measurements. In a standing position, the waist circumference was gauged at the point where the iliac crest and the lower border of the rib cage meet. All study participants received a complete clinical examination that involved measuring their height, weight, and body mass index (Kg/m2). An individual was deemed obese with a BMI of 25 Kg/m2 [14]. A Blood Pressure (BP) apparatus with mercury sphygmomanometer (Baumanometer, WA Baum Co., Copiague, NY) was used to record the BP three times at 5 minutes intervals on the right arm of the subjects in sitting condition. The average of the triplicate values was used for the analysis.

Patients with hypertension were those who were on antihypertensive drugs, who had systolic and/or diastolic blood pressure readings more than 140 mmHg, and 90 mmHg, respectively. For avoiding diurnal variation, a fasting blood sample (5 ml) was taken between 7:30 and 8:30 AM. Overnight fasting of 08-10 hours is recommended for performing the following laboratory tests: fasting plasma glucose, fasting lipid profile; test includes triglycerides (TGL), total cholesterol (TC), low-density lipoprotein cholesterol (LDL), and high-density lipoprotein cholesterol (HDL).

Commercial kits were used to perform biochemical analysis on the Fully Automated Analyzer- Beckman Coulter (USA). All patients' FPG, TC, and TGL were measured. Using the Beckman Coulter AU480 fully automatic biochemistry analyzer, FPG is measured by the Hexokinase method, TC is measured by the cholesterol oxidase phenol 4-amino antipyrine peroxidase (CHOD-PAP) method, and TGL by the glycerol phosphate oxidase-presence of peroxidase (GPO-POD) method, respectively. Although it was directly tested using readily available commercially available kits (Cholestest. LDL, Beckman Coulter, USA), the level of LDL was computed using the Friedewald formula [15]. By using the Variant fully auto machine (BioRad, USA), high-pressure liquid chromatography was used to quantify glycated hemoglobin (HbA1c). FPG levels of more than 126.0 mg/dL, HbA1c levels of more than 6.5%, insulin therapy or oral hypoglycemic medications, and/or diagnosis done by a medical physician were used to define T2DM. FPG level of more than 126.0 mg/dL with an HbA1c level of more than 6.5% detected within a year was also used to indicate newly diagnosed diabetes [16].

A spot urine sample of 25 ml was taken for the immunoturbidimetric method of estimating albuminuria in the Agappe Nepheleometer (Agappe Diagnostics Ltd., Mumbai, India). Normoalbuminuria (<30 mg/g), microalbuminuria (30 to 300 mg/g), and macroalbuminuria (>300 mg/g) were used to categorize the study subjects with T2DM. They were also sorted up into groups based on whether they received insulin, hypoglycemic pills, or just diet alone for diabetic management. Additionally noted were previous records of coronary artery disorder. Information about the consumption of fats lowering and/or antihypertensive medications was retrieved from their medical records.

Oral health habits and periodontal status

Oral health activities included brushing teeth on a regular basis and using other dental hygiene materials such as dental floss, mouth-rinse, an interdental brush, and a powered toothbrush. Dentists, who were qualified and experienced, evaluated periodontal health. Periodontitis was assessed by measuring the periodontal pocket depth (PD) using a calibrated University of North Carolina (UNC) - 15 probe and the loss of attachment (LoA) using the Community Periodontal Index (CPI) developed by the World Health Organization (WHO). A CPI score of 3 and/or 4 for PD was used to determine the presence of periodontitis. Codes 3 and 4 indicate that there were one or more sites in the index teeth with PD larger than 4 mm and 6 mm, respectively. The entire dentition was divided into six sextants represented by the index teeth 11, 16/17, 26/27, 31, 36/37, and 46/47. Scores of the index teeth often showed the periodontal health of each sextant.

The WHO recommends CPI as an epidemiological tool, and it has been widely employed for assessing PD and LoA. In each segment, the CPI score with the highest PD/LoA was recorded. In our study, periodontitis was described as having a CPI code of 3 to 4 for PD and a CPI score value of 1 to 4 for LoA. The standardized dental chart contained data on the number of teeth present, tooth mobility, and CPI code/score. The tooth mobility was scored from 0 to 3 and was classified as either present or absent (score 0).

Statistical analysis

The data were analyzed by using the SPSS Statistics for Windows, Version 26.0 (IBM Corp, Armonk, USA). The Shapiro-Wilk test was utilized to ascertain if the data were usually assigned. For continuous and categorical variables, the data were reported as mean±SD and percentages, respectively. Chi-square tests for categorical variables, the Student's t-test, and analysis of variance (ANOVA) for continuous variables were used to determine the significance of differences between groups. Tukey Post Hoc test was used for intergroup comparison among the three groups in terms of albuminuria. A p-value of ≥0.05 is considered statistically significant.

## Results

Out of 500 individuals with T2DM, 342 participants (180 males, and 162 females) had periodontitis, while 158 subjects did not (80 men and 78 women). The overall mean age was 54.7±6.6 years. The age and gender distribution between patients with and without periodontitis was determined to be statistically insignificant. Table 1 summarizes the guideline clinical parameters of the patients according to the presence of periodontitis.

**Table 1:** Clinical characteristics of study sample based on periodontal status *significant; **highly significant); LDL - Low density lipoprotein; HDL - High density lipoprotein; TG - triglycerides; CPI - Community Periodontal Index

	Periodontitis			
	Yes	No	Total	p-value
N (%)	342 (68.4)	158 (31.6)	500 (100)	
Age (years)	54.13±5.7	55.27±7.5	54.7±6.6	0.06
Gender				
Males (%)	180 (52.6)	80 (50.6)	260 (52)	0.68
Females (%)	162 (47.4)	78 (49.4)	240 (48)	
Smoker (%)	205 (59.9)	42 (26.6)	247 (49.4)	<0.001**
Alcohol intake (%)	173 (50.6)	78 (49.4)	251 (50.2)	0.80
Physical activity (%)	163 (47.7)	79 (50)	242 (48.4)	0.63
Education (≥12 years) (%)	112 (32.7)	107 (67.7)	219 (43.8)	<0.001**
Household income (>25 percentile)	97 (28.4)	95 (60.1)	192 (38.4)	<0.001**
Duration of diabetes (years)	6.52±4.8	5.48±4.93	6±4.87	0.03*
Treatment for Diabetes (%)				
Diet alone (%)	52 (15.2)	34 (21.5)	86 (17.2)	0.17
Hypoglycemic drugs (%)	244 (71.3)	101(63.9)	345 (69)	
Insulin (%)	46 (13.5)	23 (14.6)	69 (13.8)	
Newly diagnosed diabetes (%)	130 (38)	59 (37.3)	189 (37.8)	0.89
HbA1c (mmol/mol)	9.16±1.59	6.58±1.11	7.87±1.35	<0.001**
Fasting plasma glucose (mg%)	148.78±3.55	129.41±3.8	139.10±3.68	<0.001**
BMI Kg/m^2^	26.97±2.4	26.59±2.43	26.78±2.42	0.10
Systolic BP mm Hg	139.5±5.2	139±5.1	139.25±5.15	0.32
Diastolic BP mm Hg	88.5±3	88±3.01	88.25±3.01	0.08
Hypertension (%)	199 (58.2)	79 (50)	278 (55.6)	0.09
History of coronary heart disease (%)	106 (31)	46 (29.1)	152 (30.4)	0.67
Use of antihypertensive drugs (%)	106 (31)	42 (26.6)	148 (29.6)	0.98
Waist circumference cm	88.9±1.02	88.89±0.9	88.90±0.96	0.92
LDL mg/dL	112.02±3.74	108.61±4.53	110.32±4.14	<0.001**
HDL mg/dL	46.21±2.46	46.23±2.4	46.22±2.43	0.93
TG mg/dL	159.83±4.32	152.13±3.67	155.98±4	<0.001**
Total Cholesterol mg/dL	191.67±4.17	180.91±3.04	186.29±3.61	<0.001**
Use of lipid lowering drugs (%)	96 (28.1)	36 (22.8)	132 (26.4)	0.21
Urinary Albumin (Nephelometry) mg/l				
Normoalbuminuria	22.99±4.84	20.75±3.98	21.87±4.41	<0.001**
Microalbuminuria	247.76±5.8	200.81±5.04	224.29±5.42	<0.001**
Macroalbuminuria	317.53±5.61	302.46±6.13	310±5.87	<0.001**
Number of teeth present	21±6.89	25±5.26	23±6.08	<0.001**
Frequency of toothbrushing/day (%)				
1	246(71.9)	71 (44.9)	317 (63.4)	<0.001**
2	71 (20.8)	66 (41.8)	137 (27.4)	
≥3	25 (7.3)	21 (13.3)	46 (9.2)	
Use of other oral hygiene aids (%)				
Dental floss	63(18.4)	16 (10.1)	79 (15.8)	<0.001**
Interdental brush	49 (14.3)	10 (6.3)	59 (11.8)	
Mouth rinses	81 (23.7)	53 (33.5)	134 (26.8)	
None	149 (43.6)	79 (50)	228 (45.6)	
CPI Score for Loss of Attachment (%)				
1	53 (15.5)	64 (40.5)	117 (23.4)	<0.001**
2	134 (39.2)	94 (59.5)	228 (45.6)	
3	106 (31)	0	106 (21.2)	
4	49 (14.3)	0	49 (9.8)	
Mean Periodontal pocket depth, mm	5.2±4.54	2.8±2.59	4±3.57	<0.001**
Periodontal pocket depth ≥4mm (%)	112 (32.7)	0	112 (22.4)	<0.001**
Tooth mobility (%)	67 (19.6%)	0	67 (13.4)	<0.001**

In the study sample, 68.4% of participants had periodontitis. The likelihood of ever-smokers and poorer educational attainment with lower family income was statistically more in patients with periodontitis (p<0.001). The Chi-square test disclosed an insignificant difference between patients with and without periodontitis in the proportion of various diabetic therapies, anti-hypertensive/lipid-lowering medications, and the prevalence of recently identified diabetes, or hypertension. The groups did not significantly differ in terms of the cardiometabolic indicators, which include BMI, waist circumference, HDL, and systolic and diastolic blood pressure (p>0.05). However, there was an appreciable difference in FPG, HbA1c, TC, triglycerides, and LDL between persons with and without periodontitis using the t-test (p<0.001).

Patients with periodontitis had significantly lesser teeth and had DM for longer periods of time. The usage of additional oral hygiene products and the frequency with which people clean their teeth each day were revealed to be statistically different between the two groups based on their periodontal status. The mean microalbuminuria was 247.76±5.8 mg/l in patients with periodontitis, compared to 200.81±5.04 mg/l in individuals without periodontitis (p<0.001). The majority of the participants (32.7%) with periodontitis had PD ≥4 mm at one or more areas and tooth mobility (19.6%) which was statistically significant. The prevalence of microalbuminuria was statistically significant and more common in patients with periodontitis (ꭓ2=152.24; p<0.0001).

In terms of albuminuria, Table 2 compares the variables among the subjects with periodontitis.

**Table 2:** Comparison of variables between subjects with normoalbuminuria, micro-and macroalbuminuria among subjects with Periodontitis LDL - Low density lipoprotein; TG - triglycerides; CPI - Community Periodontal Index; *significant; **highly significant

N=342	Normoalbuminuria n=84	Microalbuminuria n=249	Macroalbuminuria n=9		p-value
Age	53.12±5.62	54.84±6.23	54.43±5.25		0.08
Males (%)	46 (54.8)	128 (51.4)	6 (66.7)		0.60
Females (%)	38 (45.2)	121 (48.6)	3 (33.3)		
Smoker (%)	70 (83.3)	126 (50.6)	9 (100)		0.001**
Education (≥12 years) (%)	68 (80.1)	40 (16.1)	4 (44.4)		0.001*
Household income (>25 percentile)	46 (54.8)	42 (16.9)	9 (100)		0.001**
Number of teeth present	25±6.7	19±6.94	18±7.02		<0.001**
Duration of diabetes (years)	5.46±4.58	6.9±4.27	7.2±5.55		0.03*
HbA1c	8.14±1.78	9.54±1.6	9.8±1.4		<0.001**
Fasting plasma glucose	139.64±3.07	152.53±3.68	154.17±3.9		<0.001**
LDL mg/dL	109.4±3.7	112.5±3.61	114.17±3.92		<0.001**
TG mg/dL	159.11±4.75	160.29±4.01	160.10±4.19		0.09
Total Cholesterol mg/dL	190.77±4.08	191.49±4.25	192.75±4.17		0.24
Frequency of toothbrushing/day (%)					
1	72 (85.7)	167 (67.1)	7 (77.8)	0.02*	
2	10 (11.9)	60 (24.1)	1 (11.1)		
≥3	2 (2.4)	22 (8.8)	1 (11.1)		
Other oral hygiene aids (%)					
Dental floss	15 (17.9)	47 (18.9)	1 (11.1)	<0.001**	
Interdental brush	25 (29.8)	23 (9.2)	1 (11.1)		
Mouth rinses	24 (28.6)	55 (22.1)	2 (22.2)		
None	20 (23.8)	124 (49.8)	5 (55.6)		
CPI Score for Loss of Attachment (%)					
1	31 (36.9)	21 (8.4)	1 (11.1)	<0.001**	
2	22 (26.2)	111 (44.6)	1 (11.1)		
3	21 (25)	82 (32.9)	3 (33.3)		
4	10 (11.9)	35 (14.1)	4 (44.4)		
Mean Periodontal pocket depth	4.14±4.5	5.52±4.5	5.94±4.62	0.05*	
Periodontal pocket depth ≥4mm %	15 (17.9)	89 (35.7)	8 (88.9)	<0.001**	
Tooth mobility %	20 (23.8)	40 (16.1)	7 (77.8)	<0.001**	

Normoalbuminuria, microalbuminuria, and macroalbuminuria were found in 24.6%, 72.8%, and 2.6%, respectively, in the participants with periodontitis. Participants with micro- and macroalbuminuria, had mean PD of 5.5±4.22 and 6±5.21 mm, respectively. One-way ANOVA was used to determine the statistical significance of the differences between the three groups based on albuminuria. The following characteristics showed statistically significant differences among the periodontitis subjects: smoking, education, family income, the number of teeth present, the duration of DM, LDL, the frequency of brushing, the use of other oral hygiene aids, tooth mobility, CPI score, and periodontal PD. When compared to participants with normoalbuminuria, those with micro- and macroalbuminuria had significantly higher HbA1c and FPG levels.

As indicated in Table 3, the clinical and socioeconomic characteristics of the 158 subjects free of periodontitis were contrasted in terms of albuminuria.

**Table 3:** Comparison of variables between subjects with normoalbuminuria, micro-, and macroalbuminuria among subjects without periodontitis HbA1c - glycated haemoglobin; LDL - Low density lipoprotein; TG - triglycerides; CPI - Community Periodontal Index; *significant; **highly significant

N=158	Normoalbuminuria n=128	Microalbuminuria n=22	Macroalbuminuria n=8	p-value
Age	54.95±8.5	55.59±6.5	55.27±7.5	0.06
Males (%)	61 (47.7)	12 (54.5)	7 (87.5)	0.08
Females (%)	67 (52.3)	10 (45.5)	1 (12.5)	
Smoker (%)	22 (17.2)	12 (54.5)	8 (100)	<0.001**
Education (≥12 years) (%)	91 (71.1)	14 (63.6)	2 (25)	0.02*
Household income (>25 percentile)	79 (61.7)	8 (36.4)	8 (100)	0.08
Number of teeth present	27±5.27	25±5.4	24±5.10	0.10
Duration of diabetes (years)	3.19±6.1	6.5±4	6.74±4.68	0.02*
HbA1c	6.41±1.1	6.62±1.12	6.7±1.11	0.58
Fasting plasma glucose	128.98±3.7	129±3.51	129.22±3.9	0.98
LDL mg/dL	108.16±4.8	108.65±4.63	109.02±4.17	0.82
TG mg/dL	151.27±3.71	152.38±4.12	152.74±3.19	0.28
Total Cholesterol mg/dL	180.27±2.27	180.88±3.06	181.57±3.78	0.23
Frequency of toothbrushing/day (%)				
1	61 (47.7)	6 (27.3)	4 (50)	0.46
2	50 (39.1)	13 (59)	3 (37.5)	
≥3	17 (13.3)	3 (13.6)	1 (12.5)	
Other oral hygiene aids (%)				
Dental floss	11 (8.6)	4 (18.2)	1 (12.5)	0.73
Interdental brush	8 (6.25)	1 (4.5)	1 (12.5)	
Mouth rinses	46 (35.9)	5 (22.7)	2 (25)	
None	63 (49.2)	12 (54.5)	4 (50)	
CPI Score (%)				
1 (Bleeding on probing only)	48 (37.5)	10 (45.5)	6 (75)	0.10
2 (presence of calculus)	80 (62.5)	12 (54.5)	2 (25)	
Mean gingival sulcus depth, mm	2.5±2.8	3±2.36	3±2.62	0.67

Patients with micro- and macroalbuminuria had significantly longer DM duration (p<0.05) than patients with normoalbuminuria. Smoking and educational status also revealed an appreciable difference between the three groups in subjects without periodontitis. The three categories did not vary notably in any other periodontal markers, including LDL, triglycerides, TC, brushing frequency, or family income. Table 4 illustrates the intergroup comparison of variables among subjects with albuminuria using the Tukey Post Hoc test, which showed an appreciable difference among normoalbuminuria and microalbuminuria with respect to most of the variables.

**Table 4:** Intergroup comparison of variables among subjects with normoalbuminuria, microalbuminuria, and macroalbuminuria using Tukey Post hoc test S - significant; NS - not significant; HS - highly significant; HbA1c - glycated hemoglobin; FPG - fasting plasma glucose level; LDL - low-density lipoprotein cholesterol; PD - periodontal pocket depth; DM - diabetes mellitus

	Duration of DM (P/A Periodontitis)	Number of teeth present	HbA1c	FPG	LDL	Mean PD
Normoalbuminuria Vs Microalbuminuria	S	HS	HS	HS	HS	S
Normoalbuminuria Vs Macroalbuminuria	NS	HS	HS	HS	HS	NS
Microalbuminuria Vs Macroalbuminuria	NS	NS	NS	NS	NS	NS

## Discussion

Systemic inflammation, which causes impaired pancreatic beta-cell activity, apoptosis, and insulin resistance, precedes type 2 diabetes. With more and more data pointing to heightened systemic inflammation (acute-phase and oxidative stress indicators) brought on by the circulation of periodontal organisms and their virulence factors, the link between periodontitis and diabetes is becoming biologically plausible. In the direction of the relationship between diabetes and periodontitis, AGE (Advanced Glycation Endproducts)-RAGE (Receptor for AGEs) interactions and oxidative-stress-mediated pathways offer potential molecular linkages [17].

In diabetic nephropathy condition, one of the earliest signs in patients with microalbuminuria, the bidirectional relationship is evident due to more amount of lipid accumulation leading to microvascular changes in the blood supply and thus leading to degenerative changes in the collagen of the connective tissue. This in turn is responsible for the spread of periodontal disease. It is documented that persistent microalbuminuria increases the risk of overt nephropathy by around five times over the course of 10 years in the Caucasian population with T2DM [17-19]. The total number of microalbuminuria reported in the current survey was 54.2%. In the South Indian population, Varghese et al. reported a prevalence of 36.3%. Variations in demographics, criteria for the classification of microalbuminuria, and urine collection techniques are some of the factors that may contribute to the differences in prevalence [20]. 

The albumin excretion rate normally rises steadily over several years as opposed to abruptly changing from normal to abnormal values, which occurs between 10% and 30% yearly until overt nephropathy manifests [19]. It is essential to measure microalbuminuria precisely as even slight increases in urine albumin could predict the early changes in the course of renal disorders. Therefore, the present urine albumin measurement was based on nephelometry, which was recognized as a broadly perceived standard of reference [17]. According to a study by Chen et al., the threat of generating diabetic retinopathy in T2DM can be predicted by the microalbuminuria threshold [21].

The most widely used assay for chronic hyperglycemia is the HbA1c assay. Diabetes patients with HbA1c levels of 6.5% or below have acceptable glycemic control, while those with levels over 8% incur the risk of progressing disease-related problems, like diabetic nephropathy (DN) [22]. Glycemic management is crucial in preventing diabetic microvascular sequelae. It is documented that the risk of microvascular complications decreased by 37% for every 1% decrease in the reported mean of HbA1c [23]. It is claimed that increased levels of HbA1C in T2DM are the major risk factor for the development of DN uncontrolled glycemic control [22].

With respect to normoalbuminuria, microalbuminuria, and macroalbuminuria in diabetic patients with periodontitis and/or without periodontitis, in the present study, the mean HbA1c values are demonstrated to have a significant difference in statistical analysis, which is consistent with findings noted in the prior studies [3,22-24]. Additionally, Teeuw et al. observed that individuals with periodontitis have higher HbA1c levels, and that among those with severe periodontitis, 18.1% of patients had suspected newly developed DM [25]. On the other hand, Acharya et al. identified a positive association between microalbuminuria and HbA1c; however, the data were statistically insignificant [26]. Karthikeyan et al. claimed that glycated albumin can serve as a short-term glycemic index for about 3 to 4 weeks, which will be more useful for monitoring and controlling disease progression, even though HbA1c can be an index of average plasma glucose level up to 3 months [6].

The results of the present investigation and the observations of the other studies concur that the microalbuminuric group had considerably higher FPG concentrations than the normoalbuminuric patients [7,20]. The HbA1c and FPG levels between diabetic individuals with and without periodontitis, according to Han et al., were, however, statistically insignificant [8]. In subjects with T2DM receiving periodontal care, Hayashi et al. found that there was a tendency for FPG levels to decrease [27]. The report of the current investigation was consistent with those of other research reports, which reveals that, regardless of periodontal health, the duration of diabetes had a statistically significant impact on normoalbuminuria, micro-, and macroalbuminuria in diabetic individuals [26]. The results of our investigation were consistent with the conclusive evidence that hyperglycemia and periodontal disease are related to higher levels of systemic cholesterol [7,28]. It is likely that systemic consequences of inflammatory activation presumably contribute to the correlation between periodontitis and cholesterol levels [7].

There was an appreciable variation in the number of teeth present among the individuals with and without periodontitis in the current study. The CPI, which is marked for its reproducibility and simplification, was used to identify periodontitis and evaluate its severity [1,7-9,29]. Our findings were comparable with the studies by Poplawska-Kita et al. [7], Hong et al. [29], and Han et al. [8], which demonstrate that DM notably influences all clinically assessed indicators of periodontal health. The number of teeth in each group (those with and without periodontitis) was shown to differ statistically significantly. As far as we know, this would be the initial survey to look at the impact of micro- and macroalbuminuria on the periodontal health of T2DM. Additionally, a mediated link between CKD, periodontitis, duration of diabetes, and hypertension has been shown to exist [30].

The key pathogenic mechanism connecting DM and periodontitis is a dysfunctional inflammatory response that involves immunological functioning, neutrophil activation, and cytokine biosynthesis. Prostaglandin E2, interleukin (IL)-1b, IL-6, and tumor necrosis factor (TNF)-a are among the pathways that become active in a hyperglycemic state and cause inflammation, which also has an effect on the periodontal tissues. However, prolonged subclinical systemic inflammation, a T2DM precursor, may contribute to poor glucose homeostasis. These results indicate a bidirectional interaction between hyperglycemia and periodontitis, with hyperglycemia raising the threat of periodontitis and periodontal pathology lowering glycemic status [1,28,29].

The limitations of this study are that the causal relationship between microalbuminuria and periodontitis could not be determined as it was a backward-looking cross-sectional study. The estimation of albuminuria was performed using a single urine spot sample. A multi-ethnic prospective study with a round-the-clock urine sample is necessary to control these constraints.

## Conclusions

The findings of the study suggest that T2DM patients had a greater prevalence of microalbuminuria than subjects with periodontitis and these subjects also had significantly higher HbA1c and fasting plasma glucose (FPG) levels than subjects with normoalbuminuria. In addition, subjects with periodontitis show a significant reduction in the teeth number present in terms of albuminuria. The longitudinal correlation between DM, microalbuminuria, and periodontitis could be further investigated in detail to explore possible pathways.
